# Supplementary material for: Effect of Face Masks on Blood Saturation, Heart Rate, and Well-Being Indicators in Health Care Providers Working in Specialized COVID-19 Center
Source: Int J Environ Res Public Health. 2022 Jan 26;19(3):1397. doi: 10.3390/ijerph19031397 (PMC8835197; doi:10.3390/ijerph19031397)
Supplement: Supplementary file 1 [file ijerph-19-01397-s001.zip › ijerph-1518696 - SI.pdf]

Supplementary Materials

Table S1. Depicted all participants shifts in the form of duty roster (divided into groups by sex and by professional qualification).

	10 May 2021	11 May 2021	12 May 2021	13 May 2021	14 May 2021	15 May 2021	16 May 2021	17 May 2021	18 May 2021	19 May 2021	20 May 2021	21 May 2021	22 May 2021	23 May 2021	24 May 2021	25 May 2021	26 May 2021
**Work shifts pattern ***	**Mon**	**Tue**	**Wed**	**Thur**	**Fri**	**Sat**	**Sun**	**Mon**	**Tue**	**Wed**	**Thur**	**Fri**	**Sat**	**San**	**Mon**	**Tue**	**Wed**
**00.01-3.00**																	
**3.01-6.00**																	
**6.01-9.00**																	
**9.01-12.00**																	
**12.01-15.00**																	
**15.01-18.00**																	
**18.01-21.00**																	
**21.01-24.00**																	

2	**this color is used to mark the shift of male doctors - 2 persons**
5	**this color is used to mark the shift of male nursing students - 5 persons**
3	**this color is used to mark the shift of male medical students - 3 persons**
6	**this color is used to mark the shift of female nurses - women - 6 persons**
19	**this color is used to mark the shift of female nursing students - 19 persons**
2	**this color is used to mark the shift of female medical students - 2 persons**

* The timing of 3-hour periods working with FFP2 (colored boxes),followed by 3-hour periods with FFP1 (white boxes).

Table S2. Showing an example of the week's schedule within the whole study period for representatives of all professional groups (i.e. nurses, doctors, students of nursing, and medical students).

	**10 May 2021**	**11 May 2021**	**12 May 2021**	**13 May 2021**	**14 May 2021**	**15 May 2021**	**16 May 2021**	**17 May 2021**	**18 May 2021**	**19 May 2021**	**20 May 2021**	**21 May 2021**	**22 May 2021**	**23 May 2021**	**24 May 2021**	**25 May 2021**	**26 May 2021**
	**Mon**	**Tue**	**Wed**	**Thur**	**Fri**	**Sat**	**Sun**	**Mon**	**Tue**	**Wed**	**Thur**	**Fri**	**Sat**	**San**	**Mon**	**Tue**	**Wed**
**attendings doctor (MD)**		Day shift		Day shift		Night shift		Day shift		Day shift			Day shift			Night shift	
**nurse (RN)**	Day shift		Day shift		Day shift		Night shift		Day shift		Night shift			Night shift		Day shift	
**medical student**					Day shift		Day shift		Night shift			Night shift		Day shift			Night shift
**nursing student**			Night shift			Day shift		Day shift		Day shift		Night shift		Day shift	Night shift		

Note: The shifts during which the saturation was tested were marked in green; Each shift lasts 12 hours from 7 AM to 7 PM.
